# A machine learning approach to identify predictive molecular markers for cisplatin chemosensitivity following surgical resection in ovarian cancer

**DOI:** 10.1038/s41598-021-96072-6

**Published:** 2021-08-19

**Authors:** Nicholas Brian Shannon, Laura Ling Ying Tan, Qiu Xuan Tan, Joey Wee-Shan Tan, Josephine Hendrikson, Wai Har Ng, Gillian Ng, Ying Liu, Xing-Yi Sarah Ong, Ravichandran Nadarajah, Jolene Si Min Wong, Grace Hwei Ching Tan, Khee Chee Soo, Melissa Ching Ching Teo, Claramae Shulyn Chia, Chin-Ann Johnny Ong

**Affiliations:** 1 Department of Sarcoma, Peritoneal and Rare Tumours (SPRinT), Division of Surgery and Surgical Oncology, National Cancer Centre Singapore, 11 Hospital Crescent, Singapore, 169610 Singapore; 2 Department of Sarcoma, Peritoneal and Rare Tumours (SPRinT), Division of Surgery and Surgical Oncology, Singapore General Hospital, Outram Road, Singapore, 169608 Singapore; 3 Laboratory of Applied Human Genetics, Division of Medical Sciences, National Cancer Centre Singapore, 11 Hospital Crescent, Singapore, 169610 Singapore; 4 Department of Obstetrics and Gynaecology, Division of Surgery and Surgical Oncology, Singapore General Hospital, Outram Road, Singapore, 169608 Singapore; 5 SingHealth Duke-NUS Oncology Academic Clinical Program, Duke-NUS Medical School, 8 College Road, Singapore, 169857 Singapore; 6 Institute of Molecular and Cell Biology, A*STAR Research Entities, 61 Biopolis Drive, Singapore, 138673 Singapore

**Keywords:** Cancer, Biomarkers

## Abstract

Ovarian cancer is associated with poor prognosis. Platinum resistance contributes significantly to the high rate of tumour recurrence. We aimed to identify a set of molecular markers for predicting platinum sensitivity. A signature predicting cisplatin sensitivity was generated using the Genomics of Drug Sensitivity in Cancer and The Cancer Genome Atlas databases. Four potential biomarkers (CYTH3, GALNT3, S100A14, and ERI1) were identified and optimized for immunohistochemistry (IHC). Validation was performed on a cohort of patients (n = 50) treated with surgical resection followed by adjuvant carboplatin. Predictive models were established to predict chemosensitivity. The four biomarkers were also assessed for their ability to prognosticate overall survival in three ovarian cancer microarray expression datasets from The Gene Expression Omnibus. The extreme gradient boosting (XGBoost) algorithm was selected for the final model to validate the accuracy in an independent validation dataset (n = 10). *CYTH3* and *S100A14*, followed by nodal stage, were the features with the greatest importance. The four gene signature had comparable prognostication as clinical information for two-year survival. Assessment of tumour biology by means of gene expression can serve as an adjunct for prediction of chemosensitivity and prognostication. Potentially, the assessment of molecular markers alongside clinical information offers a chance to further optimise therapeutic decision making.

## Introduction

Ovarian cancer is the seventh most common cancer in women, with over 290,000 cases diagnosed in 2018^1^. It is also the eighth most common cause of cancer death in women^2,3^. Ovarian cancer is often asymptomatic in its early stages, with approximately 60% of women having stage III or IV disease at the time of diagnosis^4^. The prognosis of ovarian cancer is poor, with a 5-year survival rate ranging from 30 to 50%^5^. Peritoneal carcinomatosis is a common feature of advanced stage epithelial ovarian carcinoma.

In the treatment of advanced-stage epithelial ovarian cancer, primary surgical cytoreduction and adjuvant chemotherapy are the preferred initial treatments. Certain patients who are poor surgical candidates due to extensively invasive disease or multiple comorbidities may undergo neoadjuvant chemotherapy. The first-line choice of chemotherapy for patients with advanced epithelial ovarian cancer includes a platinum and taxane combination^6^. Platinum agents include carboplatin and cisplatin.

While initial response rates to platinum-based chemotherapy are high at 70–80%^7^, the majority of patients with distant stage disease often relapse within two years^8^. Factors affecting tumour recurrence include initial disease stage, presence of optimal cytoreduction, CA125 levels and treatment response after chemotherapy.

Chemotherapy resistance contributes significantly to tumour recurrence. The Gynaecologic Oncology Group has stratified patients based on platinum sensitivity^9^, with platinum resistance defined as occurring in those who relapse within six months of platinum-containing therapy. Furthermore, the platinum-free interval, defined as the length of time between the last administration of the platinum agent and the appearance of disease progression, is known to correlate strongly with overall survival (OS) and progression-free survival (PFS)^10^. Despite the importance of platinum sensitivity, no predictive markers of platinum chemosensitivity in ovarian cancer have been integrated into routine clinical practice.

By identifying novel markers of chemosensitivity in ovarian cancer, we can improve therapeutic decisions by individualizing therapy for patients. Strategies for patients identified as at risk of developing platinum resistance include the early assessment of disease control and consideration of intraperitoneal or second-line chemotherapy if disease control is suboptimal. Identifying patients at risk of platinum resistance can also guide the selection of immunotherapy drugs in maintenance therapy.

We hypothesize that tumour biology plays a significant role in affecting the tumour response to chemotherapy. Understanding various genomic alterations that alter chemotherapy resistance to platinum agents would allow us to determine predictive markers of platinum resistance in ovarian cancer patients^11^. We aim to generate a panel of predictive markers of platinum chemosensitivity and to retrospectively validate these markers in tumour samples from patients who have received adjuvant chemotherapy.

## Material and methods

The study was approved by the SingHealth Centralised Institutional Review Board (Reference No.: 2015/2479). The study was conducted in compliance with all applicable SingHealth institutional policies and regulations, and the Declaration of Helsinki. Informed consent was obtained from all participants.

### Identification of potential predictive molecular markers

Publicly available datasets from the Genomics of Drug Sensitivity in Cancer (GDSC) and The Cancer Genome Atlas (TCGA) databases were used to select candidate genes for validation. The GDSC dataset consists of > 1000 genetically characterised human cell lines treated with a variety of anticancer therapeutics with matched genomic and expression data, allowing the identification of genetic features predictive of sensitivity. 39 ovarian cancer cell lines with cisplatin sensitivity data were identified. Genes were filtered based on area under the receiver operating characteristic (ROC) curve (AUC) scores ≥ 0·8, utilising gene expression to stratify cell lines into resistant vs intermediate/sensitive or sensitive vs intermediate/resistant.

We also used TCGA data, which consists of over 20,000 primary cancer and normal samples across multiple cancer types with extensive omics profiling and matched clinical information. Due to the limited number of patients treated with cisplatin (n = 19) in the ovarian cancer (OV) TCGA dataset, we decided to examine head and neck squamous cell carcinoma (HNSC). For the HNSC dataset, recurrence-free survival data were available for 518 patients. These patients were stratified into those who had been treated upfront with a platinum compound (n = 124) and those who were chemo-naive (n = 335). Patients treated with other chemotherapy agents as first-line or as subsequent therapy were excluded. Genes were filtered based on the AUC score utilising gene expression to stratify patients to early recurrence within two years vs recurrence after two years or no recurrence (platinum-treated samples, n = 36 early recurrence vs 77 late/no recurrence; chemo-naive samples, n = 34 early recurrence vs 233 late/no recurrence), with a median follow-up of two years. As recurrence is only a surrogate measure of chemosensitivity, we filtered genes limited to chemotherapy-treated patients. Genes were selected for further validation if the AUC in the platinum group was greater than the AUC in the chemo-naive group by at least 0·1 (n = 3688).

### Study population

Tissue microarrays (TMAs) containing 60 single 1-mm cores representative of ovarian cancer surgical specimens (n = 60) were purchased from TriStar Technology Group, LLC (Washington, DC, USA). These 60 surgical specimens were obtained from various accredited hospitals. Briefly, a trained pathologist identified and annotated the location of the tumour of interest on a haematoxylin and eosin (H&E) stained section of the paraffin block obtained from the hospitals. Using a TMA builder instrument, the core of interest would be punched out from the donor paraffin block and transferred to a pre-determined location on the recipient paraffin block to create the array. The constructed TMA would be lightly heated to fuse the cores with the recipient block. Of the 60 specimens, follow-up data of 59 cases were obtained. Seven of these cases consisted of patients with low-grade ovarian cancer who had undergone cytoreductive surgery and received no subsequent adjuvant therapy. One sample was excluded from the analysis because the TMA core had insufficient tissue for further analysis. The remaining cases (n = 51) consisted of patients with high-grade ovarian cancers who first underwent cytoreductive surgery. They subsequently received intravenous (IV) adjuvant chemotherapy consisting of either a carboplatin-taxol combination or carboplatin. Patients’ response to chemotherapy was assessed based on the Response Evaluation Criteria in Solid Tumours (RECIST)^12^. The change in tumour size is an objective indicator for assessing the efficiency of chemotherapy^13^. Response to chemotherapy was classified into progressive or stable disease. Patients were identified as having recurrence when there was progressive disease (PD) or when they had died of the disease or complications due to ovarian cancer. Patients with no recurrence were identified as having no evidence of disease (NED) or stable disease (SD). PD was defined as at least a 20% increase in the sum of the diameters of the target lesions. SD was identified when the sum of the diameters of the target lesions did not increase in size by more than 50% or decrease in size by more than 30%, and no new tumours developed^12^. Response was assessed after completion of neoadjuvant chemotherapy at a median duration of 181 days [IQR 151–184] from time of surgery. In the event of progressive disease, the patients were treated with carboplatin as well as a host of other chemotherapeutic agents. Their subsequent response to chemotherapy was also recorded. The clinical characteristics of the patients who received postoperative IV chemotherapy (n = 51) are further detailed in Table 1.

**Table 1:** Clinicopathological features of the patients (n = 50).

Characteristics	No	%
**Histological subtype**		
Serous papillary adenocarcinoma	41	82
Mucinous adenocarcinoma	4	8
Endometrioid carcinoma	2	4
Adenocarcinoma NOS	3	6
**Pathological stage**		
IIIA	4	8
IIIB	7	14
IIIC	27	54
IV	12	24
**Pathological T stage**		
T2a	2	4
T2b	2	4
T3a	2	4
T3b	10	20
T3c	34	68
**Pathological N stage**		
N0	27	54
N1-2	19	38
N2	4	8
**Pathological M stage**		
M0	38	76
M1	12	24
**Tumour grade**		
2	15	30
3	35	70
**IV chemo given post-surgery**		
Carboplatin-Taxol	8	16
Carboplatin	42	84
**Outcomes**		
**Recurrence**		
DOC	2	4
DOD	26	52
PD	3	6
**No recurrence**		
NED	8	16
SD	11	22

*Abbreviations* DOC, died of complications; DOD, died of disease; PD, progressive disease; NED, no evidence of disease; SD, stable disease.

### Immunohistochemistry

The TMAs were used to assess the expression of various proteins by immunohistochemistry (IHC). The antibody concentrations, optimum IHC staining conditions and antibody sources are described in Supplementary Table S1. IHC staining was performed on a Bond System (Leica Microsystems, Ltd., Milton Keynes, UK) according to the manufacturer’s recommendations. Staining intensity was stratified into 4 groups: negative (score 0), weak (score 1), moderate (score 2), and strong (score 3) for CYTH3*,* GALNT3 and S100A14 (Supplementary Fig. S1). For ERI1, scoring was binarized into negative (score 0) and positive (score 1), as the staining on the TMA only included samples with negative or weak staining despite the adequate optimisation of ERI1 antibody concentration on preliminary tissue and TMAs. The staining results were determined by 2 independent researchers (LLYT and NBS) blinded to the outcomes. For any discrepancies between the scores, a third researcher (JWST) scored the TMA sample independently to determine the final assigned score.

### Gene expression datasets

Three gene expression microarray datasets obtain from the Gene Expression Omnibus (GEO) GSE26193, GSE49997, GSE9891 had both clinical data available and the four genes present on the platform used to profile gene expression. Expression of the genes in these datasets was binarised to high or low based on the median expression and a four-gene model trained on the TMA data used to obtain a prediction score for each sample in the expression dataset. The prediction score was then assessed for prognostication of two-year overall survival. To compare to prognostication with clinical information alone, a leave one out approach was used, with the clinical information (tumour grade and tumour stage) for the other two datasets used to generate a model predicting survival in the third.

### Data processing and statistical analysis

The statistical modelling and generation of algorithms were performed using Python 3. Accuracy and ROC curve analysis were used to assess model performance. An AUC with a value of 1 indicates an ideal result, whereas values lower than 0·5 indicate an insignificant result. Generally, a value above 0·7 is satisfactory, and a value above 0·8 is excellent.

### Modelling of clinical and molecular data

A machine learning predictive model was subsequently generated to compare the combined clinical and molecular data. This process consisted of 2 aspects, namely, data input and model selection and training, as illustrated in Fig. 3a. As part of the data input, preprocessing was conducted. The protein expression of the above four genes was binarized to 0 or 1 vs 2 + (GALNT3) or 0 vs 1 + (CYTH3, ERI1, and S100A14). The clinical factors assessed were binarized as large (T3 +) vs small (T1-T2) tumours and node positive (N1 +) vs node negative (N0). Metastasis (M0 vs M1) and grade (2 vs 3) were already in a binary format. The data were split into a training set comprising 80% of the patients (n = 40) and a validation subset (n = 10) comprising 20% of the patients.

As a part of the model selection and training, typical (linear and radial support vector machine, logistic regression, K-nearest neighbour, decision tree and random forest) and boosted (AdaBoost, gradient boosting machine, XGBoost) algorithms were trained, and their performance was assessed on the training subset. Each algorithm was used to generate a predictive model. Feature selection was applied to the training dataset. The feature selection process selects the most relevant molecular markers and patient characteristics. Thresholds for each selected feature were chosen. This predictive model’s performance was subsequently assessed on the independent validation data subset. Each model was trained using five-fold cross validation with stratification to ensure consistent class distribution, as well as an internal five-fold cross validation for hyperparameter tuning utilising a 80%/20% split of training data. The learning rate of XGBoost, the final model selected, was set to 0·01, and the maximum depth of a tree was set to 3 to reduce the model complexity. To prevent overfitting, the subsample was set to 0·8. These and other hyperparameters were optimized by grid search and cross validation.

## Results

### Identification of potential predictive markers

Following a comprehensive investigation of the GDSC dataset, 41 ovarian cancer cell lines were identified. Of these, 39 had cisplatin sensitivity data and were stratified as sensitive (IC_50_ < 10 μM, n = 13), intermediate (IC_50_ 10—30 μM, n = 12) or resistant (IC_50_ > 30 μM, n = 14). A total of 248 genes were identified (n = 204 resistant, n = 31 sensitive, and n = 13 stratifying both).

A TCGA-based filter was then applied to identify genes for which stratification of patients to early recurrence within two years vs recurrence after two years or no recurrence was more apparent in platinum-treated samples (platinum-treated samples, n = 36 early recurrence vs 77 late/no recurrence; chemo-naive samples, n = 34 early recurrence vs 233 late/no recurrence). Applying this filter to the genes identified from GDSC (n = 248) narrowed the gene list to 61 genes.

**Figure 1:**
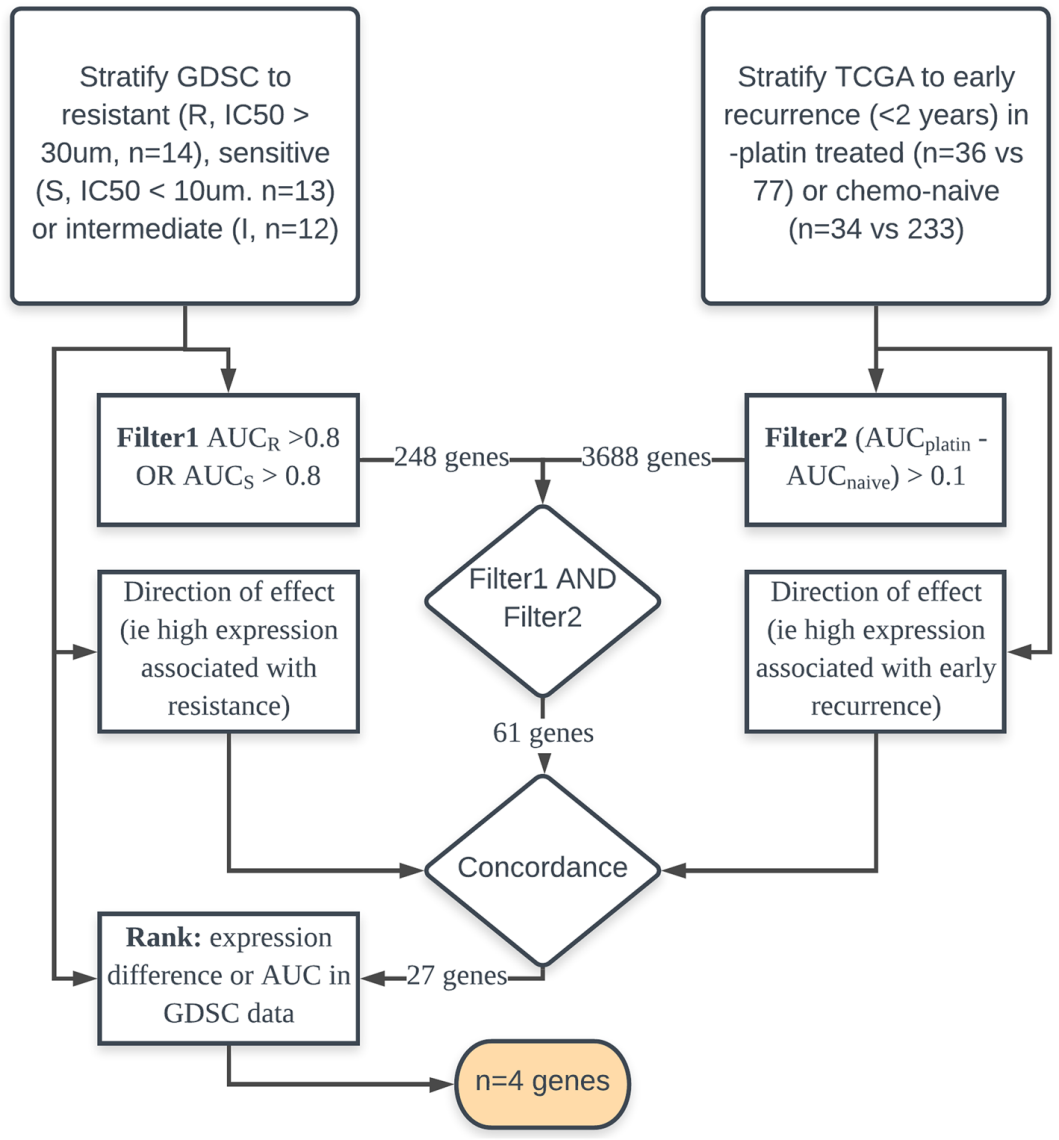
Selection of gene markers of chemoresistance.

**Figure 2:**
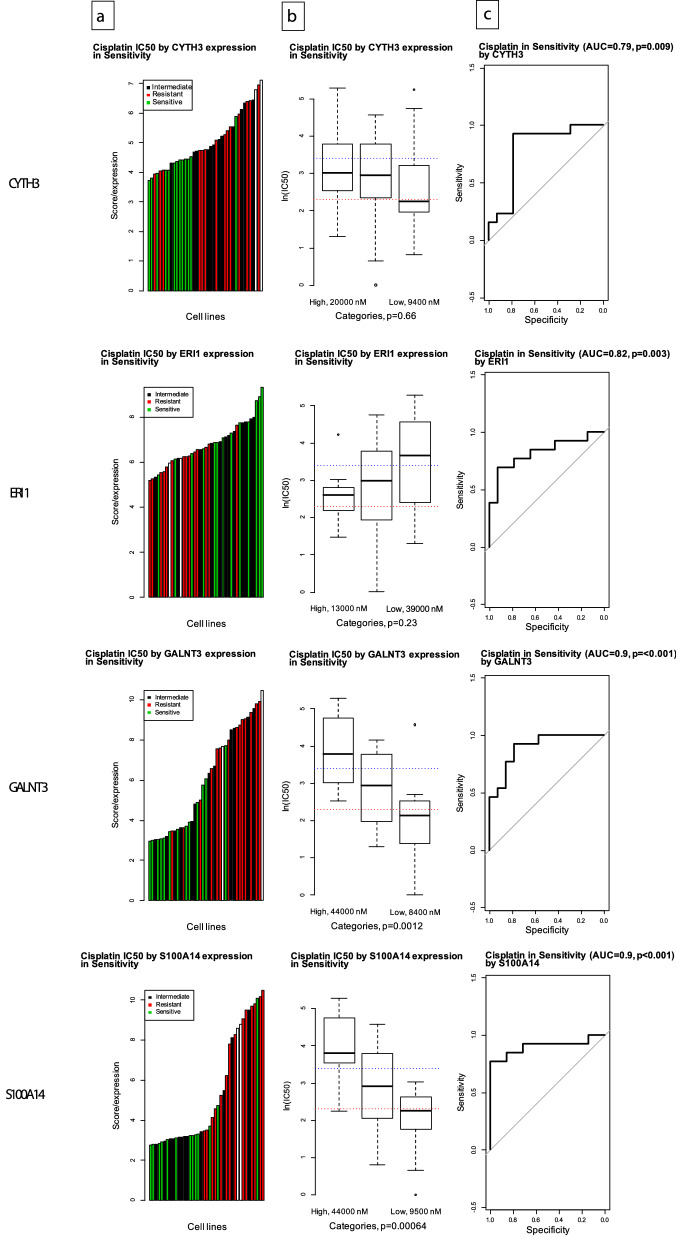
Correlation between gene expression and cisplatin IC_50_ in the identified molecular markers. (**a**) Waterfall plot representing the distribution of cisplatin sensitivity (resistant IC_50_ > 200 nM, intermediate IC_50_ > 50 nM, and sensitive IC_50_ < 50 nM) across ovarian cancer cell lines for *CYTH3*, *ERI1*, *GALNT3*, and *S100A14*. (**b**) Box plot representing IC_50_ values in cell lines grouped by expression quartiles (high = top quartile, low = bottom quartile, and medium = within interquartile range) for *CYTH3*. Dotted lines represent sensitive and resistant cut-offs of IC_50_ values for *CYTH3*, *ERI1*, *GALNT3*, and *S100A14*. (**c**) Modified ROC curve representing the ability of gene expression to correctly classify sensitive or resistant cell lines (*CYTH3* AUC = 0·79, *ERI1* AUC = 0·82, *GALNT3* AUC = 0·90, and *S100A14* AUC = 0·90), *p*-value comparing distribution of expression between sensitive and resistant cells (Mann–Whitney-Wilcoxon).

Further pragmatic filtering was performed to assess the concordance of the GDSC and TCGA data (i.e., if high expression predicted resistance in the GDSC dataset, high expression (upper quartile) should segregate patients to early recurrence in the TCGA dataset (selecting n = 27 of 61 genes)). The final list of genes was ranked by their expression difference between sensitive and resistant cell lines, and the genes were assigned points by rank according to their expression difference, using the AUC score to identify resistant or sensitive cell lines (1st place: two points, 2nd place: one point). The four genes with two points were selected for further analysis (*CYTH3, ERI1, GALNT3, and S100A14*). The process of selecting gene markers of chemoresistance is illustrated in Fig. 1. All four of these genes demonstrated a correlation between gene expression and cisplatin half maximal inhibitory concentration (IC_50_) and could potentially be used to predict chemosensitivity to cisplatin, as illustrated in Fig. 2.

**Figure 3:**
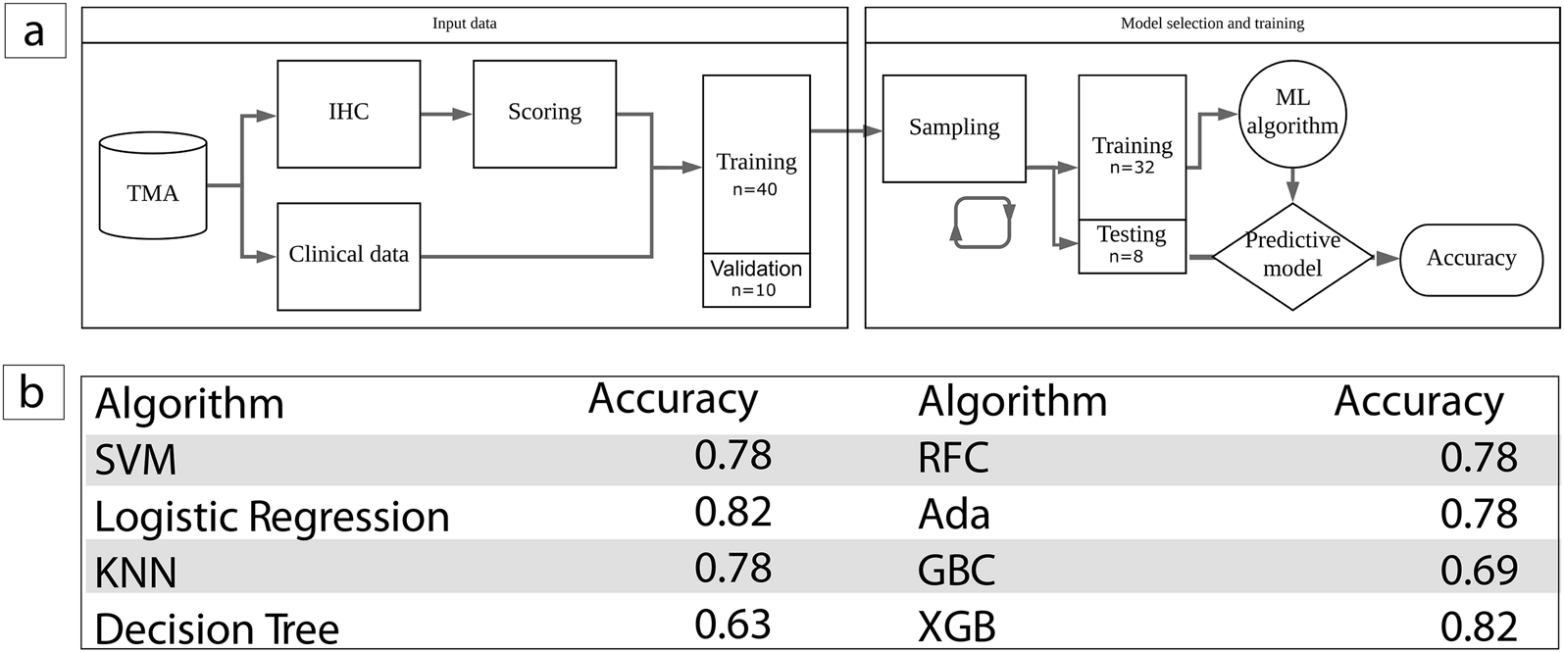
(**a**) Flow diagram illustrating the procedure to split the dataset and evaluate the model performance to classify ovarian cancer as responders or non-responders to carboplatin based chemotherapy. The tissue microarray (TMA) dataset contains cores representative of ovarian cancer surgical specimens from patients undergoing cytoreductive surgery followed by neoadjuvant chemotherapy. Data was divided into a training set (n = 40) used to select and train the model and a left out validation set (n = 10) to test the accuracy of the final model to predict chemo-sensitivity. (**b**) Accuracy of the model generated with each classifier.

A machine learning predictive model was subsequently generated to compare the combined clinical and molecular data, as illustrated in Fig. 3a. Typical (linear and radial support vector machine, logistic regression, K-nearest neighbour, decision tree and random forest) and boosted (AdaBoost, gradient boosting machine, XGBoost) algorithms were trained, and their performance was assessed on the training subset. XGBoost was selected for the final model because it had a higher accuracy than the other algorithms (Fig. 3b).

The extreme gradient boosting (XGBoost) algorithm is a tree learning algorithm that generates a decision tree to predict an outcome variable based on a series of rules concerning other variables arranged in a tree-like structure. The decision tree consists of a series of split points based on the values of the input features with the final node being a leaf giving the specific value of the output variable.

**Figure 4:**
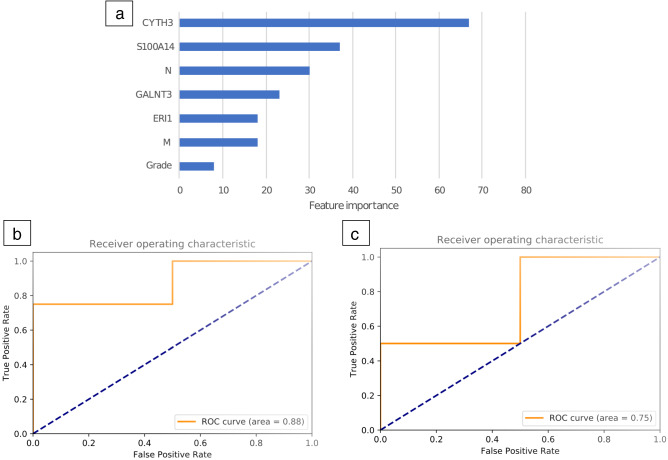
(**a**) Importance of model features in the final prediction model. Scores reflect the importance of each feature in the construction of the boosted decision trees within the model. (**b**) Performance of the XGB model in the validation dataset (10 patients) trained on 40 patients. (**c**) Logistic regression model in the validation dataset (10 patients) trained on 40 patients.

In the decision tree modelling, the model can be interpreted by looking at the importance of features across the model (Fig. 4a). Both pathological and molecular features contributed to model prediction and the features with greatest importance consisted of two of the four genes (*CYTH3* and *S100A14*), followed by N stage. The final XGBoost model tested on the validation dataset had higher performance (Fig. 4b) than logistic regression (Fig. 4c). For the XGB model, the two patients who failed first-line chemotherapy were placed 1st and 4th. For the logistic regression model, the two patients who failed first-line chemotherapy were placed 1st and 6th.

### Validation of the four-gene signature

A model consisting of the four genes trained on the TMA dataset to predict response to chemotherapy was then applied to external datasets, assessing performance of the model against the surrogate outcome of two-year survival. AUC for survival at two years was 0·67 (CI 0·55–0·79, n = 107), 0.61 (CI 0·51–0·71, n = 194) and 0·63 (CI 0·56–0·72, n = 260) in each of the three datasets respectively. Prediction using clinical information alone (tumour stage and grade), despite being trained to predict two-year survival was marginally less predictive with AUC of 0·63 (CI 0·51–0·74), 0·62 (CI 0·52–0·72), and 0·58 (CI 0·5–0·66) respectively.

## Discussion

There are no established predictors of platinum resistance that are currently utilised in routine clinical practice. Hence, our aim was to identify certain key molecular features that contribute to platinum chemotherapy resistance. This was accomplished by integrating both our molecular and clinical data to generate a predictive model to determine chemotherapy resistance to platinum agents.

We studied four genes identified using the GDSC and TCGA datasets. These genes were profiled in a TMA consisting of samples from stage III and IV ovarian cancer patients who received adjuvant chemotherapy, with response assessed in serial CT imaging using the RECIST criteria. After combining and processing the molecular and clinical data, the XGB model indicated that the features with the greatest importance in predicting chemosensitivity include the 2 markers *CYTH3* and *S100A14*, followed by nodal stage (Fig. 4a). This finding indicates that patients with higher expression levels of *CYTH3* and *S100A14* and node-positive ovarian cancer have the greatest risk of resistance to platinum chemotherapy. The feature assessment of the model suggests that tumour biology plays a significant role in resistance to platinum-based chemotherapy in ovarian cancer, as gene expression was more important for prediction than clinical information. In addition to traditional pathological factors (such as nodal stage, presence of metastasis and tumour grade), determining the presence of molecular markers (*CYTH3* and *S100A14*) may allow us to more adequately assess a patient’s risk of developing chemoresistance in ovarian cancer. The clinical utility of such prediction is additional as demonstrated by the four-gene signature also being prognostic for two-year overall survival in an additional three independent datasets. Tumours predicted as being chemoresistant should be monitored closely for response and early recurrence and considered for second-line chemotherapy once resistance is confirmed.

The exact mechanism of action of how the four markers studied contribute to chemotherapy resistance remains to be elucidated. The S100A14 protein has a role in regulating p53 protein levels, thereby playing a role in the regulation of cell survival and apoptosis. It also plays a role in the regulation of cell migration by modulating the levels of matrix metalloproteinase-2 (MMP2), a matrix protease under transcriptional control of p53. As such, intracellular S100A14 may promote cell motility and invasiveness by regulating the expression and function of MMP-2. S100A14 is also associated with tumorigenesis in colorectal cancer^14^ and breast cancer^15^. S100A14 is known to be overexpressed and associated with poor prognosis^16^ and higher recurrence rates in optimally debulked ovarian cancers^17^. Higher levels of S100A14 expression have also been correlated with resistance to platinum-based chemotherapy^18^. CYTH3 is involved in the control of Golgi structure and function, and its upregulation in hepatocellular carcinoma is associated with poor survival^19^. ERI1 is an RNA exonuclease involved in the binding to histone mRNA and in the degradation of short interfering RNAs. The roles of CYTH3 and ERI1 in ovarian cancer tumorigenesis have yet to be extensively explored in the literature.

GALNT3 protein is known to be overexpressed in high-grade serous epithelial ovarian cancer tumours and has a role in modulating post-translational modifications and metabolism pathways in ovarian cancer cells^20^. Its overexpression is correlated with poorer prognosis^21^ in advanced-stage ovarian cancer patients. GALNT3 also plays a role in membrane-associated mucin-1 (MUC1) protein stabilization. A potential mechanism for ovarian tumorigenesis involves the GALNT3-MUC1 pathway promoting cell proliferation and invasion in ovarian tumours.

Platinum resistance is a strong driving factor behind the high relapse rates of ovarian cancer, and the platinum-free interval is an important predictor of patient outcomes. At present, platinum resistance is often only determined after evidence of disease recurrence, which is seen in approximately 80% of patients with advanced stage ovarian cancer^22^. The management of relapsed disease is heavily guided by whether patients are platinum-sensitive or platinum-resistant. Patients with platinum-sensitive disease are considered for both secondary cytoreductive surgery and platinum-based chemotherapy^9^. Patients with platinum-resistant disease are given either single-agent second-line chemotherapy or a combination with bevacizumab^23^. The IHC panel and subsequent usage of the combined model will allow clinicians to better predict patient response to platinum-based chemotherapy and allow the improved individualization of chemotherapy regimens. For patients at high risk of resistance, early assessment and more intensive surveillance after surgery can help in the early detection of tumour recurrence. In terms of the choice of adjuvant therapy, patients with a higher risk of recurrence can be more readily considered for second-line chemotherapy agents beyond the standard platinum and taxane combination.

Several in vitro chemosensitivity assays, including the Chemo-FX assay and the extreme drug resistance (EDR) assay^24,25^, have been developed with a similar aim of predicting chemotherapy sensitivity. By determining the in vitro response of tumour cells, these assays aim to offer an individualised choice of chemotherapeutic agents compared to standard chemotherapy^26^. Chemo-FX is an in vitro cell culture-based drug response marker that assesses the sensitivity of ovarian tumour cells to various chemotherapeutic agents^27^. After submission of surgical specimens to a designated centre, cell cultures are formed and incubated with a panel of therapeutic drugs. While a multi-centre study has reported improved survival outcomes with the Chemo-FX assay^27^, it has not been adopted into routine clinical practice^26,28^. Reasons include a lack of prospective evaluation compared to current clinical practice and a tendency to recommend treatments that would otherwise be administered empirically^28^. In the EDR assay, fresh tumour specimens are similarly needed to identify drug resistance in tumour specimens^25^. However, its clinical relevance is limited^29^, and it is not associated with improved survival outcomes^29^.

The main strength of our study is the applicability of routine IHC stains, which can be easily adopted to individualize patient therapy compared to other chemosensitivity assays that utilize a highly specialized commercial service. In the current staging evaluation of patients with ovarian cancer, tumour samples are obtained for routine histological examination, and existing pathology services can easily adopt the IHC panel. The IHC scoring is straightforward and can be performed in the same setting when pathologists review the slides. In vitro assays such as the Chemo-FX assay require sample collection and transport to a specialist laboratory, with costs amounting to thousands of dollars per patient^30^. Therefore, the routine in vitro testing of drug sensitivity is both more difficult and costly to implement.

Furthermore, as an IHC stain, this panel can potentially be validated in a larger cohort retrospectively in future research. Currently, numerous pathology laboratories contain a tissue repository in which surgical specimens are preserved in formalin-fixed paraffin-embedded tissue blocks^31^ that allow for long-term storage and easy retrieval for research^32^. Another strength of the study is the direct assessment of chemotherapy response in our validation cohort based on the RECIST guidelines, instead of using surrogate markers of chemotherapy response such as survival or recurrence.

There are certain limitations to our study, the first being a small sample size on which to train and assess the prediction model (n = 40). A larger sample size in future studies would allow us to refine the training algorithm and improve its ability to accurately predict tumour response. Data from existing studies assessing chemotherapy assays can be used to inform sample size calculations with HR ranging from 1·5 to 2·9 depending on outcomes assessed^24^. A second limitation is with regard to tumour heterogeneity. Each donor sample was obtained from one location in the tumour. Recent studies indicate that a high degree of intratumour heterogeneity^33^ exists in advanced-stage ovarian cancer, with varying protein expression levels in various parts of the tumour. To accurately quantify protein expression via IHC, it is important to obtain biopsy samples from at least 3 different intratumour locations. The third limitation is the generalizability of the results, which is limited by the retrospective nature of the study. The donor samples were procured from various accredited hospitals. More clinical characteristics about the patient cohort (e.g., whether R0 resection was achieved) should be obtained.

In conclusion, we highlight how in addition to existing clinicopathological factors, molecular features may play a key role in determining the likelihood of platinum-based chemoresistance. Combined clinical and molecular models have the potential to identify patients at high risk of developing platinum resistance and can be straightforward to implement as part of the routine histopathological assessment of tumour specimens. Identifying such patients would allow appropriate patient management and the selection of adjuvant therapy after initial cytoreductive surgery. For patients identified as being at high risk of developing chemotherapy resistance, future studies can explore the benefit of closer surveillance or the consideration of second-line chemotherapy agents beyond the standard platinum and taxane combination. This would allow us to better individualize the chemotherapy regimen to best target tumour biology and achieve maximum response.

## Supplementary Information

Supplementary Information.

## Data Availability

The datasets generated during and/or analysed during the current study are available from the corresponding author on reasonable request. TCGA data used for gene discovery is available from The TCGA Research Network: https://www.cancer.gov/tcga. Cancer Genome Atlas Research Network, Integrated genomic analyses of ovarian carcinoma. Nature. . 2011 Jun 29;474(7353):609–15. GDSC data used for gene discovery is available from The Genomics of Drug Sensitivity in Cancer database (https://www.cancerrxgene.org/). Genomics of Drug Sensitivity in Cancer (GDSC): a resource for therapeutic biomarker discovery in cancer cells. (Nucl. Acids Res.2013 Database issue. PMID:23180760). Gene expression datasets from the Gene Expression Omnibus (GEO) can be obtained from the website [https://www.ncbi.nlm.nih.gov/geo]. Edgar R, Domrachev M, Lash AE. Gene Expression Omnibus: NCBI gene expression and hybridization array data repository. Nucleic Acids Res. 2002 Jan 1;30(1):207–10. The datasets used and references to the original manuscripts are as follows; GSE26193. Kieffer Y, Bonneau C, Popova T, Rouzier R et al. Clinical Interest of Combining Transcriptomic and Genomic Signatures in High-Grade Serous Ovarian Cancer. Front Genet 2020;11:219. PMID: 32256521. GSE49997 Pils D, Hager G, Tong D, Aust S et al. Validating the impact of a molecular subtype in ovarian cancer on outcomes: a study of the OVCAD Consortium. Cancer Sci 2012 Jul;103(7):1334–41. PMID: 22497737. GSE9891. Tothill RW, Tinker AV, George J, Brown R et al. Novel molecular subtypes of serous and endometrioid ovarian cancer linked to clinical outcome. Clin Cancer Res 2008 Aug 15;14(16):5198–208. PMID: 18698038.
