# Supplementary material for: A machine learning approach to identify predictive molecular markers for cisplatin chemosensitivity following surgical resection in ovarian cancer
Source: Sci Rep. 2021 Aug 19;11:16829. doi: 10.1038/s41598-021-96072-6 (PMC8377048; doi:10.1038/s41598-021-96072-6)
Supplement: Supplementary file 1 — Supplementary Information. [file 41598_2021_96072_MOESM1_ESM.docx]

**A machine learning approach to identify predictive molecular markers for cisplatin chemosensitivity following surgical resection in ovarian cancer**

Running head: Predicting cisplatin sensitivity in ovarian cancer by machine learning

**Authors and Affiliations**

Nicholas Brian Shannon^1,2,3^*, [nicholas.shannon@mohh.com.sg](mailto:nicholas.shannon@mohh.com.sg)

Laura Ling Ying Tan^1,2,3^*, [lauratan936@gmail.com](mailto:lauratan936@gmail.com)

Qiu Xuan Tan^1,2,3^, [qiu.xuan@nccs.com.sg](mailto:qiu.xuan@nccs.com.sg)

Joey Wee-Shan Tan^1,2,3^, [joey.tan.w.s@nccs.com.sg](mailto:joey.tan.w.s@nccs.com.sg)

Josephine Hendrikson^1,2,3^, [josephine.hendrikson@u.duke.nus.edu](mailto:josephine.hendrikson@u.duke.nus.edu)

Wai Har Ng^1,2,3^, [nmsnwh@nccs.com.sg](mailto:nmsnwh@nccs.com.sg)

Gillian Ng^1,2,3^, [gillian.ng.w.x@nccs.com.sg](mailto:gillian.ng.w.x@nccs.com.sg)

Ying Liu^1,2,3^, [liu.ying@nccs.com.sg](mailto:liu.ying@nccs.com.sg)

Xing-Yi Sarah Ong^1,2,3^, [sarah@u.duke.nus.edu](mailto:sarah@u.duke.nus.edu)

Ravichandran Nadarajah^4^, [ravichandran.nadarajah@singhealth.com.sg](mailto:ravichandran.nadarajah@singhealth.com.sg)

Jolene Si Min Wong^1,2^, [jolene.wong.s.m@singhealth.com.sg](mailto:jolene.wong.s.m@singhealth.com.sg)

Grace Hwei Ching Tan^1,2^, [grace.tan.h.c@singhealth.com.sg](mailto:grace.tan.h.c@singhealth.com.sg)

Khee Chee Soo^1,2,5^, [soo.khee.chee@singhealth.com.sg](mailto:soo.khee.chee@singhealth.com.sg)

Melissa Ching Ching Teo^1,2,5^, [melissa.teo.c.c@singhealth.com.sg](mailto:melissa.teo.c.c@singhealth.com.sg)

Claramae Shulyn Chia^1,2,5^, [claramae.chia.s.l@singhealth.com.sg](mailto:claramae.chia.s.l@singhealth.com.sg)

Chin-Ann Johnny Ong^1,2,3,5,6^**, [johnny.ong.c.a@singhealth.com.sg](mailto:johnny.ong.c.a@singhealth.com.sg)

^1^ Department of Sarcoma, Peritoneal and Rare Tumours (SPRinT), Division of Surgery and Surgical Oncology, National Cancer Centre Singapore, 11 Hospital Crescent Singapore 169610, Singapore

^2^ Department of Sarcoma, Peritoneal and Rare Tumours (SPRinT), Division of Surgery and Surgical Oncology, Singapore General Hospital, Outram Road Singapore 169608, Singapore

^3^ Laboratory of Applied Human Genetics, Division of Medical Sciences, National Cancer Centre Singapore, 11 Hospital Crescent Singapore 169610, Singapore

^4^ Department of Obstetrics and Gynaecology, Division of Surgery and Surgical Oncology, Singapore General Hospital, Outram Road Singapore 169608, Singapore

^5^ SingHealth Duke-NUS Oncology Academic Clinical Program, Duke-NUS Medical School, 8 College Road Singapore 169857, Singapore

^6^ Institute of Molecular and Cell Biology, A*STAR Research Entities, 61 Biopolis Drive, Singapore 138673, Singapore

* Equal contribution

** Corresponding author

**Corresponding Author**

Assistant Prof Chin-Ann Johnny Ong

Department of Sarcoma, Peritoneal and Rare Tumours (SPRinT), Division of Surgery and Surgical Oncology, National Cancer Centre Singapore

Laboratory of Applied Human Genetics, Division of Medical Sciences, National Cancer Centre Singapore

11 Hospital Crescent Singapore 169610

Tel: (65) 64368318

Fax: (65) 62257559

E-mail: [johnny.ong.c.a@singhealth.com.sg](mailto:johnny.ong.c.a@singhealth.com.sg)

**Supplementary Table S1.** Antibody concentration, optimum staining conditions and sources.

| Name | Concentration | Staining conditions | Source |
| --- | --- | --- | --- |
| Anti-CYTH3 Antibody | 1:200 | 20 min, Enzyme 2 | Atlas Antibodies HPA013979 |
| Anti-ERI1 Antibody | 1:100 | 20 min, Enzyme 2 | Atlas Antibodies HPA056074 |
| Anti-GALNT3 Antibody | 1:100 | 20 min, Enzyme 2 | Atlas Antibodies HPA007613 |
| Anti-S100A14 Antibody | 1:2000 | 20 min, Enzyme 2 | Atlas Antibodies HPA027613 |


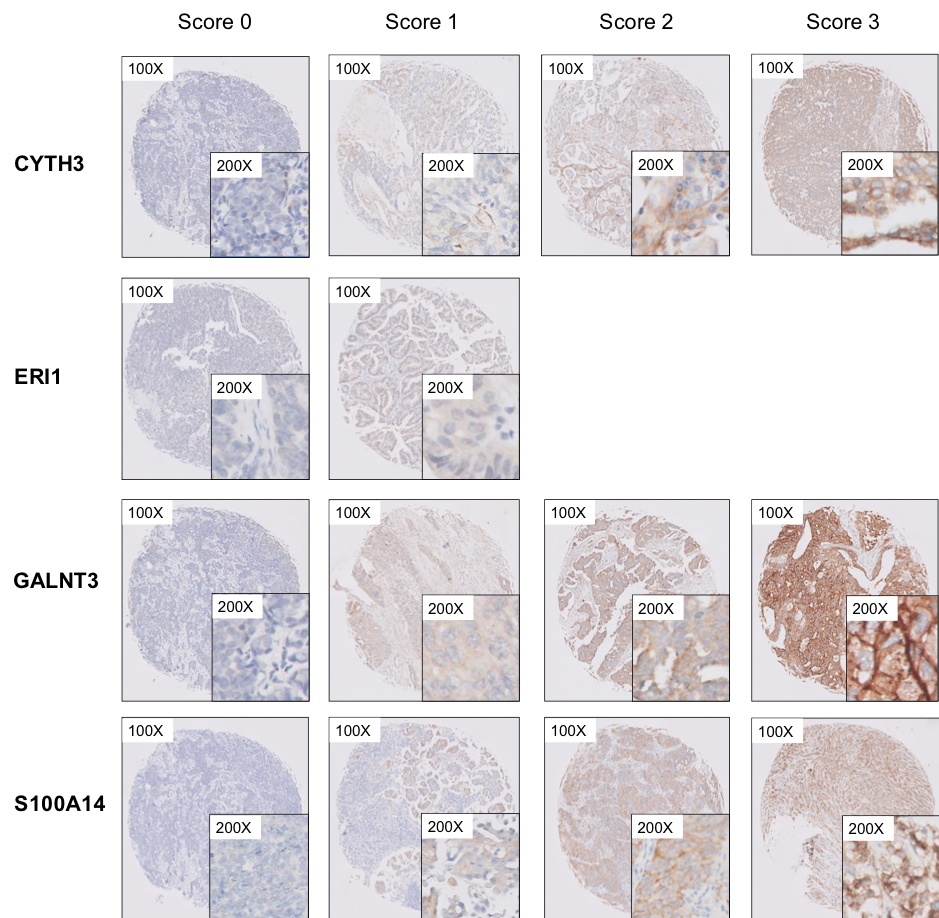


**Supplementary Figure S1.** Representative images demonstrating the scores 0 to 3 for tumour scoring seen at 100X and 200X respectively for CYTH3, GALNT3 and S100A14. Scoring for ERI1 was binarised into 0 and 1.
